# Validation of Quantitative Scores Derived From Motor Evoked Potentials in the Assessment of Primary Progressive Multiple Sclerosis: A Longitudinal Study

**DOI:** 10.3389/fneur.2020.00735

**Published:** 2020-07-24

**Authors:** Martin Hardmeier, Christian Schindler, Jens Kuhle, Peter Fuhr

**Affiliations:** ^1^Department of Neurology, University Hospital Basel, University of Basel, Basel, Switzerland; ^2^Swiss Tropical and Public Health Institute (Swiss TPH), University of Basel, Basel, Switzerland

**Keywords:** motor evoked potentials (MEP), primary progressive multiple sclerosis (PPMS), quantitative EP score, biomarker, longitudinal study

## Abstract

**Objective:** To evaluate the sensitivity to change of differently calculated quantitative scores from motor evoked potentials (MEP) in patients with primary progressive multiple sclerosis (PPMS).

**Methods:** Twenty patients with PPMS had MEP to upper and lower limbs at baseline, years 1 and 2 measured in addition to clinical assessment [Expanded Disability Status Scale (EDSS), ambulation score]; a subsample (*n* = 9) had a nine-hole peg test (NHPT) and a timed 25-foot walk (T25FW). Quantitative MEP scores for upper limbs (qMEP-UL), lower limbs (qMEP-LL), and all limbs (qMEP) were calculated in three different ways, based on *z*-transformed central motor conduction time (CMCT), shortest corticomuscular latency (CxM-sh), and mean CxM (CxM-mn). Changes in clinical measures and qMEP metrics were analyzed by repeated-measures analysis of variance (rANOVA), and a factor analysis was performed on change in qMEP metrics.

**Results:** Expanded Disability Status Scale and ambulation score progressed in the rANOVA model (*p* < 0.05; *post-hoc* comparison baseline–year 2, *p* < 0.1). Lower limb and combined qMEP scores showed significant deterioration of latency (*p* < 0.01, MEP-LL_CxM-sh: *p* < 0.05) and in *post-hoc* comparisons (baseline–year 2, *p* < 0.05), qMEP_CxM-mn even over 1 year (*p* < 0.05). Effect sizes were higher for qMEP scores than for clinical measures, and slightly but consistently higher when based on CxM-mn compared to CxM-sh or CMCT. Subgroup analysis yielded no indication of higher sensitivity of timed clinical measures over qMEP scores. Two independent factors were detected, the first mainly associated with qMEP-LL, the second with qMEP-UL, explaining 65 and 29% of total variability, respectively.

**Conclusions:** Deterioration in qMEP scores occurs earlier than EDSS progression in patients with PPMS. Upper and lower limb qMEP scores contribute independently to measuring change, and qMEP scores based on mean CxM are advantageous. The capability to detect subclinical changes longitudinally is a unique property of EP and complementary to clinical assessment. These features underline the role of EP as candidate biomarkers to measure effects of therapeutic interventions in PPMS.

## Introduction

Development of therapies in primary progressive multiple sclerosis (PPMS) is hampered by the fact that detecting disease progression by clinical assessment needs considerable sample sizes and follow-up time to be meaningful (1, 2). Biomarkers allowing shorter multicenter clinical trials in small patient groups are not well-established (3, 4), and several candidate biomarkers have been proposed, including evoked potentials (EPs) (5).

Evoked potentials yield complementary information to clinical assessment as they are closely related to demyelination and measure subclinical changes, which may transform only later into clinical disability. Animal models have not only shown close correlations between demyelination and latency delay (6), but also between the recovery of delayed latencies with remyelination, bidirectionally paralleled by clinical function (7, 8). Several clinical studies have reported that scores from multimodal EP are predictive of disease course in relapsing and progressive multiple sclerosis (MS) [review in (9)], and short-term test–retest variability is reasonably low for quantitative EP scores (qEPS) (10). Longitudinal EP studies, which evaluate sensitivity to change of EP scores, are scarce in PPMS. In one small study, a multimodal qEPS deteriorated after 6 months, whereas the Expanded Disability Status Scale (EDSS) became significantly worse only after 12 months (11).

Motor evoked potentials (MEPs) to upper and lower limbs are an essential part of a multimodal EP assessment. Out of several measures derived from MEPs, latency is most closely linked to abnormal signal conduction in the corticospinal tract and a robust and easily registered MEP component (12). For diagnostic purposes, it is recommended to use the central motor conduction time (CMCT), which is specific for abnormalities in central signal conduction (13). However, test–retest reliability of CMCT is lower as compared to corticomuscular latency (CxM) (10), making CxM probably better suited to monitor disease course, provided that peripheral nerve disease has been excluded beforehand.

From a pathophysiological point of view, both latency delay and variability of MEP onset are features of disturbed signal propagation (14). In MS, onset latencies have been shown to be significantly more variable than in healthy controls and independent of latency delay (15). Moreover, the dispersion of MEP responses has been included in a semiquantitative EP scoring system (16). To account for onset variability and latency in one number, we currently calculated the mean CxM (CxM-mn), which is close to the shortest CxM (CxM-sh) in case of low variability and markedly longer in the case of high variability.

In the current study, we aim to scrutinize the MEP component of the multimodal qEPS regarding sensitivity to change in an independent sample of patients with PPMS and to determine the optimal way of its calculation.

For this purpose, we calculated qMEP scores based on CMCT, CxM-sh, and CxM-mn for upper limbs (qMEP-UL), lower limbs (qMEP-LL), and the combination of both (qMEP); evaluated longitudinal change of these nine qMEP metrics, as well as of clinical measures; and performed a factor analysis to determine the contribution of the different qMEP metrics to measuring change in latencies.

## Methods

### Subjects

Twenty subjects with PPMS had MEP and clinical assessment at baseline years 1 and 2. Inclusion criteria were aged between 18 and 65 years and a primary progressive disease course as defined in the 2017 revisions of the McDonald criteria (17). Exclusion criteria comprised contraindications to MEP recording (epilepsy, moveable metal implants, pacemaker, pregnancy), inability to provide informed consent, and the presence of other diseases than MS interfering with MEP recording. All patients gave written informed consent in accordance with the Declaration of Helsinki.

### Clinical Assessment

Patients were examined at least annually at our MS center by certified physicians using the EDSS (18) as defined in Neurostatus (19). Neurostatus includes an ambulation score ranging from 0 (unrestricted) to 12 (restricted to bed or chair, EDSS 8.0), which differs from the EDSS in a more granular representation of EDSS steps 6.0 and 6.5, where the ambulation scores are 5 to 7 and 8 to 9, respectively, taking walking distance and kind of walking aid used into account (see Supplemental Material). However, EDSS steps 0 to 4.0 are only represented as ambulation scores 0 to 1. All EDSS scores were checked for congruency with rating of functional systems and ambulation.

In a subsample, a nine-hole peg test (NHPT) as a timed measure of dexterity and a timed 25-foot walk (T25FW) as a timed measure of ambulation were available. They were performed according to the standards described in the Multiple Sclerosis Functional Composite [*z*-transformed relative to the NMSS sample (20)].

### MEP Assessment

All MEPs to upper and lower limbs were recorded in our laboratory (Department of Neurology Hospital of the University of Basel) according to internal standards closely following the recommendations of the International Federation of Clinical Neurophysiology (IFCN) (13). Our clinical protocol is optimized for reproducibility and time efficiency using parasagittal stimulation with a round coil (MagProCompact, C-100, coil diameter 12.5 cm; Magventure Farum, Denmark; or Magstim 200, coil diameter 14 cm; The Magstim Company; Whitland, Wales, Great Britain) for upper and lower limbs at 80 to 100% stimulator output. Facilitation is achieved by slight contraction of the target muscles (m. abductor digiti minimi for upper limbs, m. tibialis anterior for lower limbs); for the spinomuscular latency, magnetic stimulation over the spine (cervical vertebra 7; lumbar vertebra 5) is applied. Cortical stimulation comprises eight stimuli (four coil side A, four coil side B), spinal stimulation four stimuli (two with coil side A, two with coil side B), recorded bilaterally resulting in eight cortical and four spinal responses per side.

All MEP curves were exported from the recording machine and uploaded to EPMark, a software tool for standardized EP reading. All curves were rated by a single rater (M.H.); follow-up curves were rated in comparison to baseline examinations to reduce inconsistencies due to curve rating.

Motor evoked potentials were analyzed for each side and limb and calculated in three ways based on the shortest CxM (CxM-sh), the mean CxM (CxM-mn), and the CMCT (difference between the CxM-sh and shortest spinomuscular latency). Mean CxM was calculated only if at least three of eight responses were available. In one patient, the unrecordable year 1 values of lower limb MEP were replaced by the baseline values. All CxM and CMCT values were *z*-transformed and corrected for height in lower limbs (see Supplemental Material), *z*-values from left and right sides were averaged to yield a one number score for upper limbs (qMEP-UL), lower limbs (qMEP-LL), and all limbs (qMEP) for each mode of calculation.

### Statistical Analysis

Statistical analyses were conducted using SPSS Version 22 (IBM Corporation, Armonk, NY, USA). Repeated-measures analysis of variance (ANOVA) was used to compare means of qMEP metrics and clinical assessments longitudinally expressing the degree of temporal variation by partial η^2^. *Post-hoc* comparisons between pairs of time points were conducted using Bonferroni correction. For each MEP index and each patient, the three consecutive measurements were summarized by an average linear slope [slope = (*x*(year 2) – *x*(year 0))/2] and a non-linear trend [trend = (*x*(year 2) – 2^*^x(year 1) + *x*(year 0))]. A factor analysis using principal component analysis followed by Varimax rotation was run on the two parameters across the nine qMEP metrics. For a sensitivity analysis of the NHPT, a paired *t*-test was run on all subjects with at least two assessments of the NHPT (see Supplemental Material).

## Results

Subjects had a mean age of 51.3 years (SD = 7.9) and a disease duration of 8.2 (SD = 6.7) years. The mean time between baseline and year 1 as well as year 2 assessments was 0.99 (SD = 0.12) and 2.1 (SD = 0.14) years, respectively. Median EDSS at baseline was 3.75 (range = 2.0–6.5), and median ambulation score 1 (0–9). Mean and standard deviation (SD) of qMEP metrics are given in the fifth column of Table 1.

**Table 1:** Analysis of longitudinal change in EDSS, ambulation and qMEP scores.

		***F*_**(2, 38)**_**	***p*-value**	**Effect size**	**Mean y0**	**Change y1-y0**	**Change y2-y1**	**Change y2-y0**
**Clinical**	EDSS	3.278	**<0.05**	0.147	3.9 (1.2)	0.18 (−0.23 to 0.58)	0.33 (−0.25 to 0.90)	0.50 (−0.06 to 1.06) ^∧^
	Ambulation	4.499	**<0.05**	0.191	1.9 (2.2)	0.70 (−0.11 to 1.51)	0.55 (−0.55 to 1.65)	1.25 (−0.07 to 2.57) ^∧^
**qMEPUL**	CMCT	1.773	n.s.	0.085	3.62 (2.85)	0.05 (−1.04 to 1.13)	0.75 (−0.57 to 2.07)	0.80 (−0.53 to 2.13)
	CxM_sh	1.575	n.s.	0.077	5.13 (2.95)	0.04 (−071 to 0.79)	0.49 (−0.43 to 1.40)	0.52 (−0.39 to 1.43)
	CxM_mn	2.178	n.s.	0.103	4.33 (3.87)	0.15 (−0.57 to 0.86)	0.51 (−0.35 to 1.37)	0.66 (−0.35 to 1.67)
**qMEPLL**	CMCT	5.468	**<0.01**	0.223	5.24 (5.04)	0.98 (−0.42 to 2.38)	0.86 (−0.56 to 2.29)	**1.84 (0.29 to 3.40)** ^*^
	CxM_sh	4.588	**<0.05**	0.195	7.04 (5.23)	0.74 (−0.25 to 1.74)	0.54 (−0.55 to 1.62)	**1.28 (0.03 to 2.54)** ^*^
	CxM_mn	5.832	**<0.01**	0.235	7.67 (7.05)	0.75 (−0.13 to 1.62)	0.61 (−0.38 to 1.60)	**1.36 (0.12 to 2.60)** ^*^
**qMEP**	CMCT	6.285	**<0.01**	0.249	4.43 (3.61)	0.51 (−0.37 to 1.40)	0.81 (−0.23 to 1.84)	**1.32 (0.29 to 2.36)** ^*^
	CxM_sh	5.422	**<0.01**	0.222	5.92 (3.64)	0.39 (−0.22 to 1.00)	0.51 (−0.24 to 1.27)	**0.90 (0.12 to 1.69)** ^*^
	CxM_mn	7.530	**<0.01**	0.284	6.00 (5.09)	**0.61 (0.07 to 1.15)** ^*^	0.56 (−0.20 to 1.33)	**1.17 (0.17 to 2.17)** ^*^

*Univariate repeated measures ANOVA (n = 20) and *post-hoc* paired comparisons for EDSS and ambulation score as well as qMEP scores calculated from upper limbs (qMEP-UL), lower limbs (qMEP-LL), and the combination of both (qMEP) based on central motor conduction time (CMCT), shortest cortico-muscular latency (CxM_sh), and mean CxM (CxM_mn). QMEP-scores are given as the sum of z-transformed latencies divided by the number of limbs examined. For all variables, F-values, p-values (linear contrast), and effect sizes are given along with their mean values and standard deviations (SD) at baseline (y0), and their mean changes between different years, with 95% confidence intervals (95%CI)*.

∧ *p < 0.1*;

* *p < 0.05, with Bonferroni correction for multiple comparisons. Significant values are given in bold*.

At baseline, a subsample of patients had assessments of NHPT (*n* = 13) and T25FW (*n* = 9), of whom nine subjects had NHPT and T25FW at all three time points.

**Figure 1:**
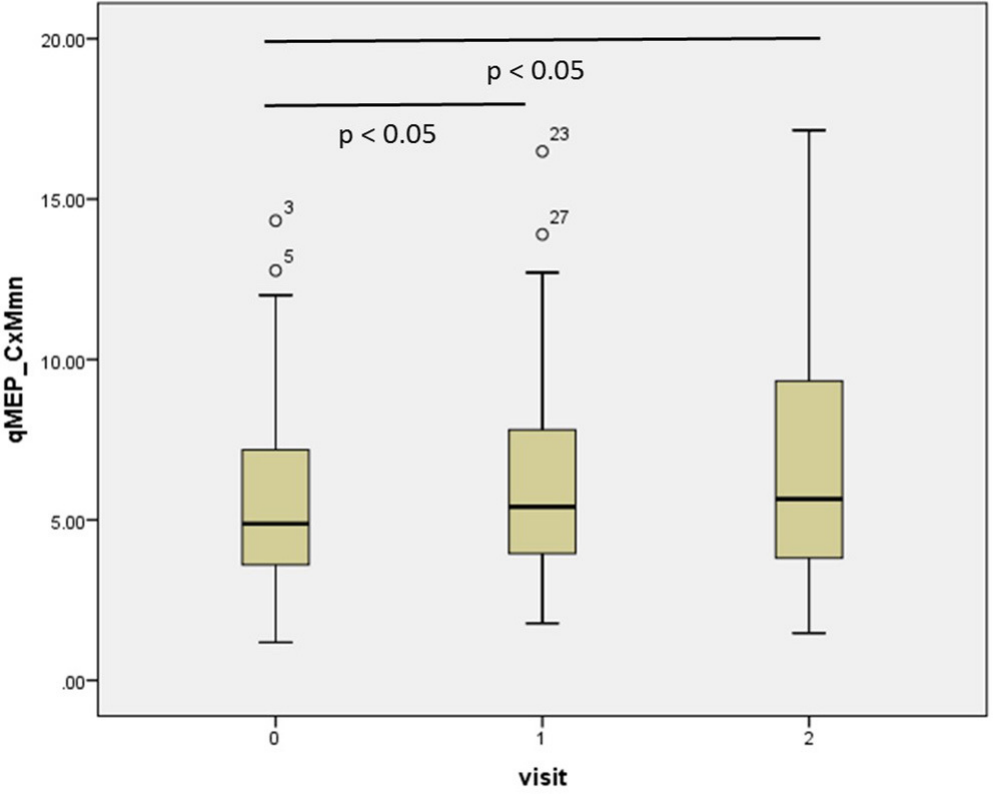
Boxplot diagram showing the distribution of the combined quantitative MEP score based on the mean corticomuscular latency (qMEP_CxM-mn) at baseline, years 1 and 2. *P*-values are given for pairwise *post-hoc* comparison after Bonferroni correction.

The results of the repeated-measures ANOVA are given in Table 1; *p*-values relate to the linear contrasts. EDSS and ambulation score progressed over time (*p* < 0.05), with a non-significant change over the 2 year period (*p* < 0.1 after Bonferroni correction). Latency increased significantly in qMEP-LL (*p* < 0.01 for CxM-mn and CMCT, *p* < 0.05 for CxM-sh) and combined qMEP scores (all *p* < 0.01), and the increase in the qMEP-CxM_mn score being statistically significant even in the first year (*p* < 0.05), as depicted in Figure 1. Effect sizes were higher in qMEP-LL and combined qMEP scores than in clinical assessments, and highest in scores based on CxM_mn. QMEP-UL did not significantly change over time.

Subgroup analysis (Table 2) in subjects (*n* = 9) with complete assessments of the NHPT and the T25FW showed a similar pattern. Whereas, changes in clinical measures were not significant (ambulation: *p* < 0.1; others *p* > 0.1), qMEP-LL_CxM-mn, qMEP_CxM-mn, and qMEP_CxM-sh showed statistically significant deterioration with highest effect sizes for measures calculated from CxM-mn. The sensitivity analysis of the NHPT (Supplemental Material) based on subjects with a baseline and a year 2 examination (*n* = 12) yielded a comparable non-significant change for the NHPT (*p* = 0.06) and the qMEP-UL_CxM-mn (*p* = 0.1).

**Table 2:** Subgroup analysis of longitudinal change in T25FW and NHPT.

		***F*_**(2, 16)**_**	***p*-value**	**Effect size**	**Mean y0**	**Change y1-y0**	**Change y2-y1**	**Change y2-y0**
**Clinical**	EDSS	2.266	n.s.	0.147	3.94 (1.13)	−0.28 (−0.85 to 0.29)	0.78 (−0.53 to 2.09)	0.50 (−0.81 to 1.81)
	Ambulation	3.653	<0.1	0.191	1.89 (1.83)	0.44 (−0.58 to 1.46)	1.44 (−0.87 to 3.75)	1.89 (−0.97 to 4.75)
	zT25FW	2.048	n.s.	0.204	9.18 (8.12)	−0.05 (−0.45 to 0.34)	−1.27 (−3.82 to 1.23)	−1.32 (−4.18 to 1.55)
	zNHPT	1.065	n.s.	0.118	−0.74 (1.05)	0.07 (−0.24 to 0.38)	−0.26 (−0.99 to 0.47)	−0.19 (−0.76 to 0.37)
**qMEPUL**	CMCT	1.727	n.s.	0.178	4.00 (5.21)	0.32 (−1.43 to 2.06)	1.11 (−1.44 to 3.66)	1.43 (−1.45 to 4.31)
	CxM_sh	1.726	n.s.	0.177	3.43 (3.52)	0.22 (−0.99 to 1.43)	0.77 (−0.99 to 2.53)	0.99 (−1.00 to 2.99)
	CxM_mn	2.056	n.s.	0.204	4.83 (3.49)	0.44 (−0.74 to 1.61)	0.66 (−0.91 to 2.22)	1.10 (−0.96 to 3.14)
**qMEPLL**	CMCT	2.474	n.s.	0.236	7.00 (6.5)	0.11 (−2.34 to 2.55)	1.52 (−1.15 to 4.19)	1.63 (−0.66 to 3.92)
	CxM_sh	2.668	n.s.	0.250	4.65 (4.71)	0.14 (−1.54 to 1.83)	1.11 (−0.84 to 3.06)	1.26 (−0.48 to 3.00)
	CxM_mn	4.123	**<0.05**	0.340	6.43 (5.04)	0.34 (−1.17 to 1.85)	1.27 (−0.46 to 2.99)	1.61 (−0.45 to 3.66)
**qMEP**	CMCT	3.558	<0.1	0.308	5.63 (5.46)	1.47 (−0.48 to 3.43)	−0.06 (−1.71 to 1.60)	1.42 (−0.62 to 3.45)
	CxM_sh	4.489	**<0.05**	0.359	4.07 (3.61)	−0.01 (−1.02 to 1.00)	1.06 (−0.12 to 2.23)	1.05 (−0.40 to 2.49)
	CxM_mn	5.629	**<0.05**	0.413	5.25 (3.49)	0.60 (−0.28 to 1.49)	1.03 (−0.25 to 2.31)	1.63 (−0.42 to 3.68)

*Univariate repeated measures ANOVA and post-hoc paired comparisons on all patients with complete T25FW and NHPT (n = 9) for EDSS, ambulation score, z-transformed timed 25 foot walk (zT25FW) and z-transformed nine hole peg test (zNHPT) as well as qMEP scores calculated from upper limbs (qMEP-UL), lower limbs (qMEP-LL), and the combination of both (qMEP) based on central motor conduction time (CMCT), shortest cortico-muscular latency (CxM_sh), and mean CxM (CxM_mn). For all variables, F-values, p-values (linear contrast), and effect sizes are given along with their mean values and standard deviations (SD) at baseline (y0), and their mean changes between different years with 95% confidence intervals (95%CI). Significant values are given in bold. All post-hoc comparisons were non-significant*.

**Figure 2:**
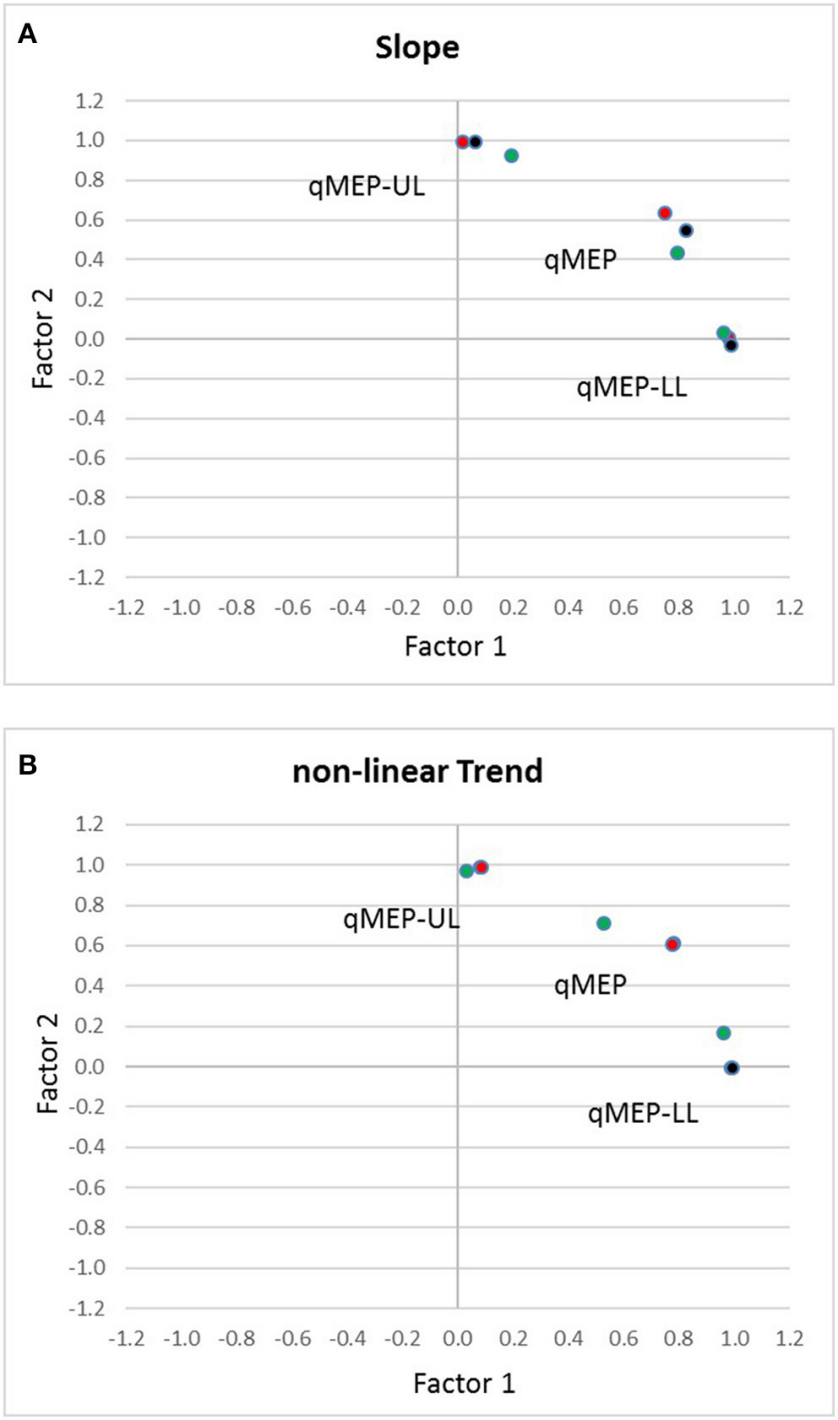
Diagrams show the separation of quantitative MEP scores for upper (qMEP-UL) and lower limbs (qMEP-LL), as well as the combination of both (qMEP) along two independent dimensions obtained by factor analysis after Varimax rotation using **(A)** individual slopes (linear trends) **(B)** individual deviations from linearity (non-linear trends). The *x*- and *y*-coordinates of the variables are defined by their loadings on the first and second factors. Colors represent the different ways of calculating the scores from central motor conduction time (CMCT; red), shortest corticomuscular latency (CxM-sh; black), and mean CxM (CxM-mn; green).

Factor analysis (Table 3) showed that qMEP-UL and qMEP-LL provide complementary information for the detection of longitudinal change in MEP onset latency, regardless whether the parameter of change was the linear slope or a non-linear trend (Figure 2). The first factor was mainly determined by qMEP-LL, and the second factor mainly by qMEP-UL explaining 65 and 29% of total variability. The combined qMEP scores load on both factors in a balanced way.

**Table 3:** Factor analysis of longitudinal change in qMEP metrics.

		**Linear contrasts**		**Non-linear contrasts**	
		**Factor 1**	**Factor 2**	**Factor 1**	**Factor 2**
**Model**	Eigenvalue	5.876	2.610	5.945	2.608
	Explained variance %	65.3	29.0	66.1	29.0
**Factor loadings**					
qMEPUL	CMCT	0.065	0.986	0.089	0.983
	CxM_sh	0.196	0.920	0.081	0.986
	CxM_mn	0.019	0.988	0.035	0.969
qMEPLL	CMCT	0.980	0.001	0.965	0.166
	CxM_sh	0.991	−0.031	0.994	−0.008
	CxM_mn	0.965	0.029	0.991	−0.010
qMEP	CMCT	0.797	0.433	0.777	0.605
	CxM_sh	0.829	0.545	0.782	0.607
	CxM_mn	0.749	0.634	0.529	0.709

*Factor analysis of linear and quadratic contrasts of temporal change in qMEP metrics defined at the individual patient level using principal component and Varimax rotation. Eigenvalues, explained variance and factor loadings are given. Both individual contrasts revealed two independent dimensions (factors). The loading matrices show that the two factors of each contrast are largely determined by the lower and upper limb measurements, respectively*.

## Discussion

In the current study, we examined 20 patients with primary progressive MS longitudinally over 2 years to scrutinize qMEP scores regarding sensitivity to change and to determine the optimal way of calculating the qMEP. In parallel to clinical progression as measured by EDSS and ambulation score over 2 years, lower limbs and combined qMEP scores indicated significant deterioration of latency delays with higher effect sizes than the EDSS and ambulation score. Differences between differently calculated qMEP scores were small, albeit scores based on mean CxM had highest effect sizes throughout, and only the combined qMEP score based on mean CxM showed a significant deterioration already in the first year. Moreover, in a subgroup analysis, timed clinical assessments did not show higher sensitivity than qMEP scores. Two independent factors were detected, the first mainly associated with qMEP-LL, the second one with qMEP-UL, explaining 65 and 29% of total variability, respectively. Upper and lower limb qMEPs contribute to the combined qMEP score in a balanced way.

Our main finding is that increases in latency delays over 2 years, as measured by lower limb and combined qMEP scores, were stronger in terms of effect size than increases in disability as measured by EDSS. Moreover, significant deterioration in the first year was observed in the combined qMEP based on mean CxM, but not in any of the clinical parameters. This result replicates the principal findings of a previous study in PPMS (11) in an independent sample of patients and is in line with several EP studies showing deterioration of EP scores over time in samples with relapsing remitting MS, as well as samples with relapsing and progressive MS [review in (9)]. In the former PPMS study, a multimodal qEPS changed already after 6 months, whereas the EDSS deteriorated only after 1 year (11). The higher temporal dynamics are most likely due to the faster clinical progression in the previous sample. Additionally, the applied multimodal qEPS includes motor, somatosensory, and visual EP, which probably increases the sensitivity to change. As individual patients are likely to deteriorate in different functional systems at different pace, a multimodal EP score is more likely to capture changes than a single modality. However, it remains to be determined whether the different EP modalities are equally sensitive to change.

In a recent cross-sectional study, MEPs from upper limbs only have been proposed as an outcome measure in clinical trials in patients with progressive forms of MS (21). The authors argue that lower limb MEPs are frequently absent and do not contribute to measuring deterioration. However, patients had considerable disability with a mean EDSS of 5.8, and the majority had a secondary progressive MS. In contrast, the current longitudinal analysis in less disabled patients with primary progressive MS clearly shows the high contribution of lower limb involvement to disease progression. Furthermore, upper and lower limb qMEP scores contribute independently to measuring disease progression. These results favor the use of a combined qMEP score, at least in patients with comparable disease characteristics and disability.

Variability of onset latencies is a physiological phenomenon and most likely due to short-term fluctuations in cortical and spinal excitability (13, 22). In MS, reliability of signal conduction is reduced in demyelinated tracts (14) due to less accurate temporal summation at the convergence of corticospinal axons in the spinal motoneuron. Significantly increased variability of MEP onset as quantified by the mean consecutive difference between several stimuli has been found in patients with MS independent of latency delay (15). However, in the current factor analysis, we could not detect an independent contribution of mean CxM, indicating that onset variability may not add to detection of change. Our approach may have been less sensitive than the mean consecutive difference, which, on the downside, poses other problems when used in a score, as it is an additional metric and a relative measure.

The slightly but consistently better performance of mean CxM over shortest CxM and CMCT may be related to its statistical properties with higher test–retest reliability (10) because an averaged response is a more robust estimate than a maximal response. However, the closer relationship to pathophysiology by inclusion of the variability of the onset latencies may also play a role.

To increase the sensitivity of clinical assessment for detecting progression, a combination of the EDSS with timed examinations as the NHPT and T25FW has been proposed in progressive MS (23, 24). There are only a few studies that compared EP with timed clinical assessment. Upper limb MEP correlated with the NHPT (21) and lower limb MEP with T25FW (25) cross-sectionally. Balance problems were more closely related to tibial somatosensory EP than to lower limb MEP (26). In the current study, we had only a small subsample to compare timed clinical measures to qMEP scores longitudinally. In these patients, we found no evidence indicating that NHPT or T25FW was superior to qMEP scores. However, the present sample size is too small to draw firm conclusions. Larger scaled studies are needed to better characterize the comparative sensitivity to change of timed clinical assessments and EP scores from different modalities.

Generally, clinical assessment and EP differ in their content validity. Expanded Disability Status Scale, NHPT, and T25FW measure global clinical function, dexterity, and walking capability, respectively (18, 27, 28). They are influenced by day-to-day fluctuations in performance, as well as imprecision of the clinical rating. Moreover, compensatory mechanisms may allow patients still to function, although marked damage has already occurred (29). In contrast, EPs are closely linked to the pathophysiology of disturbed signal conduction (7, 8, 14), regardless of whether delayed responses are clinically symptomatic or remain subclinical. The transformation of such subclinical pathology into clinical disability is the most likely explanation for the prognostic power of multimodal EP assessment [review in (9)].

The stimulation protocol used in the current and in previous studies of our group (11, 30) differs from the recommendations of the IFCN regarding the determination of the resting motor threshold (RMT) (13). The standard method (31) is time consuming and requires the application of up to 75 stimuli. A proposed optimization of the method needs handling of additional software (32). The use of a standard stimulation intensity of 80 to 100% of stimulator output with a non-focal round coil is a pragmatic approach, which is time-efficient and easy to standardize. It induces a supramaximal cortical stimulation in nearly all subjects with a small overall number of stimuli. Moreover, it is probably near the recommended stimulation intensity of 140 to 170% RMT taking into account that RMT is higher in MS (33), and on average at 70% of stimulator output according to one study with progressive MS (34).

The main limitation of the current study is its small sample size, which greatly reduces the generalizability of the current findings. Furthermore, NHPT and T25FW were only available in a subgroup, rendering the comparison between these timed assessments and qMEP scores preliminary. However, our main results replicate the findings of a previous study in an independent sample (11), corroborating the validity of the use of EP for measuring change.

## Conclusions

The current study confirms a finding of our previous study demonstrating that deterioration in a qEPS occurs earlier than clinical progression as measured by the EDSS in patients with primary progressive MS. Both upper and lower limb qMEP scores contribute independently to measuring change, and qMEP scores calculated from mean CxM showed slightly higher effect sizes than scores calculated from shortest CxM or CMCT. In most target populations, a combined qMEP score based on upper and lower limbs mean CxM is therefore a reasonable choice. The previously used multimodal qEPS may even increase the sensitivity to change.

The capability to detect subclinical change is a unique property of EP and complementary to clinical examination. Evoked potential assessment may even open a window within which therapeutic effects can be quantified, before a clinical effect is detectable. These features and the current results underline the role of EP as a candidate biomarker to measure effects of therapeutic interventions in PPMS.

## Data Availability Statement

The datasets generated for this study will not be made publicly available. The raw data supporting the conclusions of this article will be made available by the authors, without undue reservation, to any qualified researcher.

## Ethics Statement

The studies involving human participants were reviewed and approved by formerly Ethikkommission beider Basel, EKBB; currently Ethikkommission Nordwest- und Zentralschweiz, EKNZ; which approved the study protocols (EKBB 206/13; EKBB 161/12). The patients/participants provided their written informed consent to participate in this study.

## Author Contributions

MH, CS, JK, and PF contributed to the study design, data acquisition, data analysis, data interpretation, manuscript drafting, and approved the final version. All authors contributed to the article and approved the submitted version.

## Conflict of Interest

The authors declare that the research was conducted in the absence of any commercial or financial relationships that could be construed as a potential conflict of interest.
