# Supplementary material for: Validation of Quantitative Scores Derived From Motor Evoked Potentials in the Assessment of Primary Progressive Multiple Sclerosis: A Longitudinal Study
Source: Front Neurol. 2020 Jul 24;11:735. doi: 10.3389/fneur.2020.00735 (PMC7393441; doi:10.3389/fneur.2020.00735)
Supplement: Supplementary file 1 [file Data_Sheet_1.docx]

**Supplementary material**

**Ambulation score**

(Version 04/10.2, according to https://www.neurostatus.net/scoring/index.php)

Unrestricted ambulation means the patient is able to walk a distance without assistance that is regarded as normal, compared with healthy individuals of similar age and physical condition. In this case, the EDSS step can be anything between 0 and 5.0, depending on the FS scores. Fully ambulatory means at least 500 meters of ambulation without assistance, but not unrestricted. The EDSS step can be anything between 2.0 and 5.0, depending on the FS scores. In this case, the pyramidal and / or cerebellar FS must be > 2 to reflect this „restriction“ of ambulation. If ambulation is < 500 meters, the EDSS step must be > 4.5 depending on the walking ranges provided by the ambulation score (see next page) and combination of FS scores. EDSS steps 5.5 to 8.0 are exclusively defined by the ability to ambulate and type of assistance required, or the ability to use a wheelchair. If assistance is needed, the definitions of EDSS steps 6.0 or 6.5 include both a description of the type of assistance required when walking and the walking range. Assistance by another person is equivalent to bilateral assistance.

Definitions:

0 Unrestricted

1 Fully ambulatory

2 > 300 meters, but < 500 meters, without help or assistance (EDSS 4.5 or 5.0)

3 > 200 meters, but < 300 meters, without help or assistance (EDSS 5.0)

4 > 100 meters, but < 200 meters, without help or assistance (EDSS 5.5)

5 Walking range < 100 meters without assistance (EDSS 6.0)

6 unilateral assistance, > 50 meters (EDSS 6.0)

7 bilateral assistance, > 120 meters (EDSS 6.0)

8 unilateral assistance, < 50 meters (EDSS 6.5)

9 bilateral assistance, > 5 meters, but < 120 meters (EDSS 6.5)

10 Uses wheelchair without help; unable to walk 5 meters even with aid, essentially restricted to wheelchair; wheels self and transfers alone; up and about in wheelchair some 12 hours a day (EDSS 7.0)

11 Uses wheelchair with help; unable to take more than a few steps; restricted to wheelchair; may need some help in transferring and in wheeling self (EDSS 7.5)

12 essentially restricted to bed or chair or perambulated in wheelchair, but out of bed most of day; retains many self-care functions; generally has effective use of arms (EDSS 8.0)

**Normative values used for z-transformation.** Values are mean (SD).

MEP-UL: CMCT: 6.0ms (0.9) (Claus 1990)

CxM-sh/CxM-mn: 19.7ms (1.3) (Hess in Stoehr et al. 2005);

MEP-LL: CMCT: height*0.19-17.783 (1.17) (Hess in Stoehr et al. 2005)

CxM-sh/CxM-mn: height*0.308 - 23.587 (1.6) (Hess in Stoehr et al. 2005)

Reference:

Claus D. Central motor conduction: method and normal results. Muscle Nerve. 1990; 13:1125.

Hess C. Motorisch Evozierte Potentiale. In: Stoehr, Dichgans, Buettner, Hess (eds). *Evozierte Potentiale*, 4th ed, Heidelberg: Springer-Verlag, 2005, p 570, 574

**Sensitivity analysis for nine-hole-peg test (pairwise comparisons)**

| a) |  | y0 (SD) | change y1 - y0 (95% CI) | p-value |
| --- | --- | --- | --- | --- |
| clinical | zNHPT | -0.83 (1.03) | 0.02 (-0.22-0.26) | **n.s.** |
|  |  |  |  |  |
| qMEPUL | CMCT | 4.17 (4.94) | 0.0 (-1.37 - 1.37) | **n.s.** |
|  | CxM-sh | 3.45 (3.32) | 0.0 (-0.94 - 0.94) | **n.s.** |
|  | CxM-mn | 4.86 (3.29) | 0.25 (-0.64 - 1.14) | **n.s.** |

| b) |  | y0 (SD) | change y2 - y0 (95% CI) | p-value |
| --- | --- | --- | --- | --- |
| clinical | zNHPT | -0.61 (1.13) | -0.31 (-0.65 - 0.02) | .064 |
|  |  |  |  |  |
| qMEPUL | CMCT | 4.32 (4.63) | 1.17 (-0.50 - 2.84) | .152 |
|  | CxM-sh | 3.79 (3.37) | 0.81 (-0.35 - 1.96) | .152 |
|  | CxM-mn | 5.21 (3.45) | 0.95 (-0.21 - 2.12) | .100 |

Tab. S1. Two-sided paired t-test for comparison of z-transformed NHPT values (zNHPT), and upper limb qMEP scores (qMEP-UL) based on central motor conduction time (CMCT), shortest cortico-muscular latency (CxM-sh) and mean CxM (CxM-mn) between a) baseline and year 1 (n=10), and b) baseline and year 2 (n=12). Mean values (SD) are given for baseline (y0), along with mean change between a) baseline and year 1 and b) baseline and year 2 (lower and upper limits of 95% confidence interval), as well as the p-value is given for.
